# Prediction of lncRNA-disease associations by integrating diverse heterogeneous information sources with RWR algorithm and positive pointwise mutual information

**DOI:** 10.1186/s12859-019-2675-y

**Published:** 2019-02-19

**Authors:** Xiao-Nan Fan, Shao-Wu Zhang, Song-Yao Zhang, Kunju Zhu, Songjian Lu

**Affiliations:** 1 Key Laboratory of Information Fusion Technology of Ministry of Education, School of Automation, Northwestern Polytechnical University, 127 West Youyi Road, Xi’an, 710072 Shaanxi China; 2 Department of Biomedical Informatics, University of Pittsburgh, 5607 Baum Blvd, Pittsburgh, PA 15206 USA; 3 The First Affiliated Hospital and Clinical Medicine Research Institute, Jinan University, Guangzhou, China

**Keywords:** Long noncoding RNA, Disease, lncRNA-disease association, Heterogeneous network, Random walk with restart algorithm

## Abstract

**Background:**

Long non-coding RNAs play an important role in human complex diseases. Identification of lncRNA-disease associations will gain insight into disease-related lncRNAs and benefit disease diagnoses and treatment. However, using experiments to explore the lncRNA-disease associations is expensive and time consuming.

**Results:**

In this study, we developed a novel method to identify potential lncRNA-disease associations by Integrating Diverse Heterogeneous Information sources with positive pointwise Mutual Information and Random Walk with restart algorithm (namely IDHI-MIRW). IDHI-MIRW first constructs multiple lncRNA similarity networks and disease similarity networks from diverse lncRNA-related and disease-related datasets, then implements the random walk with restart algorithm on these similarity networks for extracting the topological similarities which are fused with positive pointwise mutual information to build a large-scale lncRNA-disease heterogeneous network. Finally, IDHI-MIRW implemented random walk with restart algorithm on the lncRNA-disease heterogeneous network to infer potential lncRNA-disease associations.

**Conclusions:**

Compared with other state-of-the-art methods, IDHI-MIRW achieves the best prediction performance. In case studies of breast cancer, stomach cancer, and colorectal cancer, 36/45 (80%) novel lncRNA-disease associations predicted by IDHI-MIRW are supported by recent literatures. Furthermore, we found lncRNA LINC01816 is associated with the survival of colorectal cancer patients. IDHI-MIRW is freely available at https://github.com/NWPU-903PR/IDHI-MIRW.

**Electronic supplementary material:**

The online version of this article (10.1186/s12859-019-2675-y) contains supplementary material, which is available to authorized users.

## Background

Long non-coding RNAs (lncRNAs) are the biggest part of non-coding RNAs with at least 200 nucleotides and no observed potential to encode proteins [1, 2]. To date, 15,778 lncRNA genes and 27,908 lncRNA transcripts have been annotated in human genome by the GENCODE v27. Increasing evidences have revealed that lncRNAs have key roles in gene regulations, affecting cellular proliferation, survival, migration and genomic stability [3–7]. Therefore, there is no surprise that mutation and dysregulation of lncRNAs could contribute to the development of various human complex diseases [8–10], such as HOTAIR in breast cancer [11] and MALAT1 in early-stage non-small cell lung cancer [12]. On the other hand, lncRNAs can drive many important cancer phenotypes through their interactions with other cellular macromolecules including DNA, protein, and RNA [4]. For example, lncRNA PCGEM1 and PRNCR1 are associated with androgen receptor in prostate cancer cells [6]. And lncRNA PTCSC3 could be a tumor suppressor in thyroid cancer cells by interacting with miR-574-5p [13].

In recent years, the number of experimentally verified lncRNA-disease associations is gradually increasing. Several databases for lncRNA functions and disease associations have been published, such as LncRNAdb [14], LncRNADisease [15], Lnc2Cancer [16] and NONCODE [17]. However, known lncRNA-disease associations still involve a small part of lncRNAs and diseases. Computational methods have been developed to predict the potential lncRNA-disease associations that can be used as candidates for biological experiment verifications, which would greatly reduce the experiment cost and save time for finding new lncRNA-disease associations. Existing computational methods can mainly be categorized into machine learning-based methods [18–29] and network-based methods [30–41]. The machine learning-based methods, such as LRLSLDA [18], LDAP [26], and MFLDA [27], have been developed to predict the potential lncRNA-disease associations. LRLSLDA [18] combined optimal classifiers in lncRNA space and disease space into a single classifier to predict lncRNA-disease associations based on lncRNA expression profiles and known lncRNA-disease associations. But how to combine the classifiers reasonably needs to further study. LDAP [26] employed two lncRNA similarity measures and five disease similarity measures to calculate lncRNA similarities and disease similarities, respectively, then used the bagging SVM to predict lncRNA-disease associations. However, this method suffered from fusing multiple similarities effectively. Fu et al. [27] developed a lncRNA-disease associations prediction model (MFLDA) with matrix factorization by integrating seven relational data sources between six object types (e.g. lncRNAs, miRNAs, genes, Gene Ontology, Disease Ontology, and drugs). Yet, MFLDA can only predict the potential lncRNA-disease associations which share both lncRNAs and diseases with known associations in training set.

The network-based methods, such as RWRlncD [30], RWRHLD [32], KATZLDA [33] and GrwLDA [40], use lncRNA-disease association, disease similarity, lncRNA similarity, and other molecular similarity to construct the lncRNA similarity networks, or lncRNA-disease heterogeneous network, then implement global network models (such as random walk and various propagation algorithms) to predict potential lncRNA-disease associations [10]. RWRlncD [30] constructed a lncRNA similarity network based on known lncRNA-disease associations, i.e., each lncRNA in their network has at least one known lncRNA-disease association, for predicting potential lncRNA-disease associations. So, the major limitation of RWRlncD is that it cannot predict lncRNA-disease associations for lncRNAs and diseases without any known lncRNA-disease associations. RWRHLD [32] calculated lncRNA similarities and disease similarities based on crosstalk between lncRNAs and miRNAs and directed acyclic graph in the disease ontology, respectively. One weakness of RWRHLD is that lncRNAs interacting with similar miRNAs do not always mean related with similar diseases, and only a small fraction of lncRNA-miRNA interactions is used [25]. KATZLDA [33] integrated lncRNA expression similarity, lncRNA functional similarity, Gaussian interaction profile kernel similarity for diseases and lncRNAs, disease semantic similarity, and known lncRNA-disease associations to build a lncRNA-disease heterogeneous network, then used KATZ algorithm to calculate potential association probability of each lncRNA-disease pair. GrwLDA [40] introduced a global network random walk method to predict potential lncRNA-diseases association by integrating disease semantic similarity, lncRNA functional similarity and known lncRNA-disease associations. Overall, the results of existing network-based methods show that integrating diverse lncRNA-related and disease-related information can boost the prediction accuracy of the lncRNA-disease association. However, most existing methods are limited to a small number of lncRNAs and diseases. For example, the network built in RWRHLD involves 697 lncRNAs and 126 diseases, while the network built in GrwLDA just involves 78 lncRNAs and 113 diseases. In addition, most existing methods calculate the lncRNA/disease similarities only on those that have at least one known lncRNA-disease association.

To address the aforementioned issues (or limitations) and further improve the prediction accuracy, we proposed a novel network-based method, namely IDHI-MIRW, to predict the potential lncRNA-disease associations by constructing a large-scale lncRNA-disease heterogeneous network with Random Walk with Restart (RWR) algorithm and the positive pointwise mutual information (PPMI). Instead of constraining lncRNA and disease on those with at least one known lncRNA-disease association, IDHI-MIRW calculates the lncRNA similarities for all the lncRNAs involved in lncRNA expression profiles, lncRNA-miRNA interactions, and lncRNA-protein interactions, and also calculates the diseases similarities for all the diseases involved in disease ontology, disease-miRNA associations, and disease-gene associations. Then, IDHI-MIRW uses the RWR algorithm on each similarity network to capture network topological structural features for measuring the lncRNA/disease topological similarity through the PPMI. By integrating the lncRNA/disease topological similarity, and introducing the known lncRNA-disease association information, a large-scale lncRNA-disease heterogeneous network is built. Finally, the random walk with restart on heterogeneous network (RWRH) algorithm [42] is applied on the lncRNA-disease heterogeneous network to predict the potential lncRNA-disease associations. The computational results show that IDHI-MIRW cannot only better predict the known lncRNA-disease associations, but also can effectively predict the potential lncRNA-disease associations, providing more candidates for experimental verification. Most of the new predicted lncRNA-disease associations are supported by recent literatures. By analyzing nine unvalidated lncRNAs, we found that six lncRNAs were differentially expressed in corresponding cancers. We also found that lncRNA LINC01816 is associated with the survival of colorectal cancer patients, which provides evidence that this lncRNA is disease-related.

## Results

In this section, we first introduced the evaluation method and metrices for evaluating the performance of the IDHI-MIRW method. Then, we compared our IDHI-MIRW method with other existing state-of-the art methods on a small-scale lncRNA-disease heterogeneous network, explored the predictive power of IDHI-MIRW on a large-scale lncRNA-disease heterogeneous network, and discussed the effect of different parameters. In the end, we analyzed several predicted potential lncRNA-disease associations with our IDHI-MIRW.

### Evaluation method and metrices

The leave-one-out cross validation (LOOCV) test method was used to evaluate the performance of the IDHI-MIRW method. In LOOCV test method, each known lncRNA-disease association in the dataset is singled out in turn as a test sample, and the remaining lncRNA-disease associations are used as training samples. That is, for a given disease *d*_*i*_, each known lncRNA associated with *d*_*i*_ is left out in turn as a test sample, and corresponding association edge between test lncRNA and *d*_*i*_ is removed, and the remaining lncRNAs associated with *d*_*i*_ are considered as training samples.

The area under the receiver operating characteristic (ROC) curve (AUC) and the area under the precision-recall (PR) curve (AUPR) were used as evaluation metrices in our experiments. The ROC curve is the plot of the true-positive rate (TPR, or Recall) versus the false-positive rate (FPR) at different rank cutoffs. The PR curve is the plot of the ratio of true positives among all positive predictions for each given recall rate.

### Comparison with other methods

**Fig. 1:**
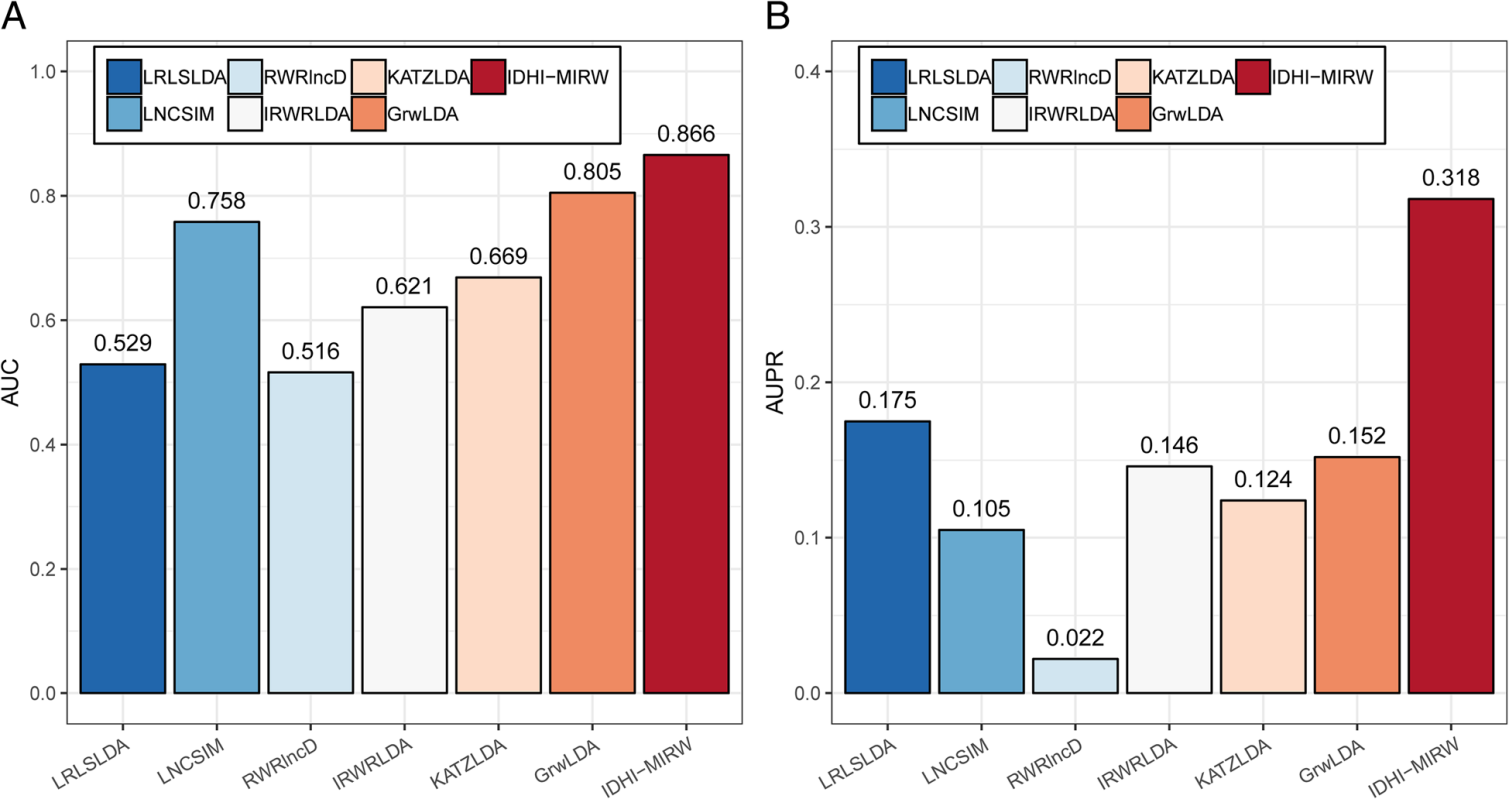
Results of IDHI-MIRW, LRLSLDA, LNCSIM, RWRlncD, IRWRLDA, KATZLDA and GrwLDA on a small-scale lncRNA-disease heterogeneous network in LOOCV test. **a** AUC values. **b** AUPR values

We compared our IDHI-MIRW method with other six state-of-the-art methods of LRLSLDA [18], LNCSIM [19], RWRlncD [30], IRWRLDA [34], KATZLDA [33] and GrwLDA [40] on the small-scale lncRNA-disease heterogeneous network (HNet_S_) which contains 362 lncRNAs, 370 diseases, and 2169 known lncRNA-disease associations. Most existing methods often built this small-scale lncRNA-disease heterogeneous network in which each lncRNA (or disease) has at least an associated disease (or lncRNA) to predict the potential lncRNA-disease associations. LRLSLDA [18] and LNCSIM [19] adopt the semi-supervised learning frameworks with Laplacian regularized least squares. RWRlncD [30], IRWRLDA [34], KATZLDA [33] and GrwLDA [40] are the network-based methods. All methods were executed on a win10 system pc with i7–6700 CPU and 16.0G memory. Figure 1 shows the AUC and AUPR values of IDHI-MIRW and other six methods. IDHI-MIRW achieved a better performance than other six methods in terms of AUC and AUPR. The AUC of IDHI-MIRW is 0.866, which is 0.337, 0.108, 0.350, 0.245, 0.197 and 0.061 higher than that of LRLSLDA, LNCSIM, RWRlncD, IRWRLDA, KATZLDA and GrwLDA, respectively. The AUCPR of IDHI-MIRW is 0.318, which is 0.143, 0.213, 0.296, 0.172, 0.194 and 0.166 higher than that of LRLSLDA, LNCSIM, RWRlncD, IRWRLDA, KATZLDA and GrwLDA, respectively. The recall values of seven methods at different rank cutoffs are listed in Table 1, from which we can see that the recall value of IDHI-MIRW is higher than that of other six existing methods at 10, 20, 50, and 100 ran cutoff. These results show that our IDHI-MIRW can effectively predict the lncRNA-disease associations.

**Table 1:** Recalls of seven methods at different cutoffs on a small-scale lncRNA-disease heterogeneous network in LOOCV test

	Top10	Top20	Top50	Top100
LRLSLDA	0.320	0.406	0.447	0.462
LNCSIM	0.217	0.402	0.595	0.704
RWRlncD	0.005	0.012	0.038	0.161
IRWRLDA	0.273	0.344	0.432	0.563
KATZLDA	0.251	0.382	0.554	0.661
GrwLDA	0.276	0.437	0.652	0.721
IDHI-MIRW	0.461	0.623	0.766	0.845

**Fig. 2:**
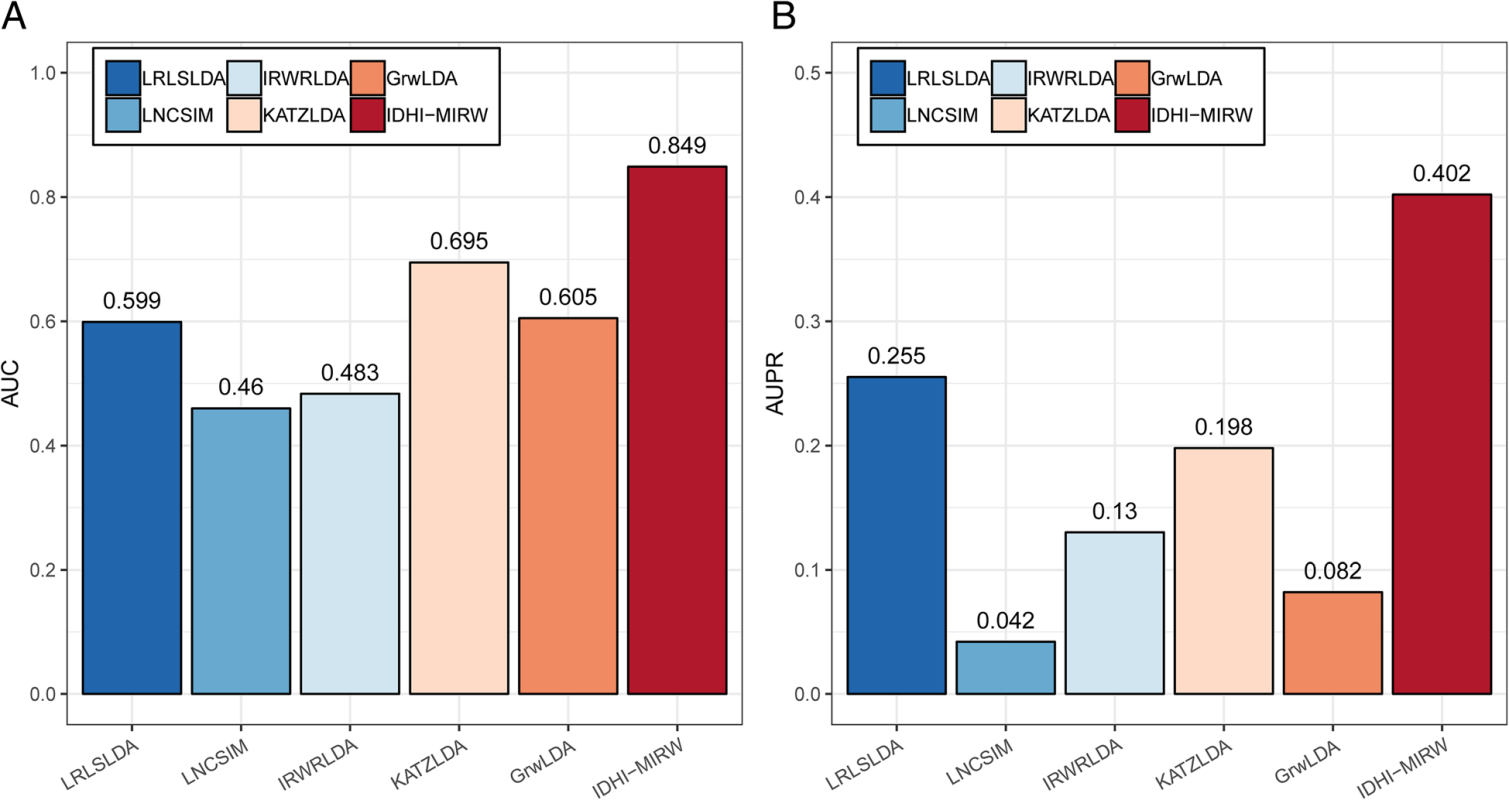
Prediction results for diseases without any known disease association information. **a** AUC values. **b** AUPR values

To further evaluate the performance of IDHI-MIRW for predicting the associated lncRNAs for new diseases without any known lncRNA association information, we removed all the known lncRNA associations for the query disease in the small-scale lncRNA-disease heterogeneous network. Due to RWRlncD implemented the RWR algorithm on an lncRNA similarity network, we just compared our IDHI-MIRW method with other five methods of LRLSLDA, LNCSIM, IRWRLDA, KATZLDA and GrwLDA for predicting the associated lncRNAs of the query diseases. The comparison results are shown in Fig. 2, which shows that our IDHI-MIRW method can better predict the associated lncRNAs for the new disease than other existing prediction methods.

### Effectiveness of introducing multiple information sources

In order to illustrate the effectiveness of introducing multiple information sources, we collected 7637 lncRNAs and 6453 diseases from EMBL-EBI (E-MTAB-5214), starBase v2.0 [43], NPInter v3.0 [44], RAID v2.0 [45], Diseases ontology [46], HMDD v2.0 [47], and DisGeNet [48] to construct a large-scale lncRNA-disease heterogeneous network (HNet_L_) by introducing 2169 known lncRNA-disease associations, then implemented our IDHI-MIRW method on HNet_L_. Additional files 1 and 2 provided the data processing procedure for lncRNAs and diseases. The results of IDHI-MIRW on HNet_S_ and HNet_L_ heterogeneous networks in LOOCV test are listed in Table 2, from which we can see that introducing more lncRNAs and diseases can effectively improve the predictive performance of IDHI-MIRW and can predict the potential lncRNAs/diseases for new disease/lncRNA without any known disease/lncRNA association information. All these results show that IDHI-MIRW can obtain a more reliable performance for predicting lncRNA-disease associations.

**Table 2:** Results of IDHI-MIRW on the small-scale lncRNA-disease heterogeneous network and large-scale lncRNA-disease heterogeneous network in LOOCV test

Network	AUC	AUPR	Recall			
			Top10	Top20	Top50	Top100
HNet_S_	0.866	0.318	0.461	0.623	0.766	0.845
HNet_L_	0.952	0.350	0.449	0.614	0.790	0.851

### Effectiveness of using the topological similarity network to construct the lncRNA-disease heterogeneous network

In order to evaluate the effectiveness of using the topological similarity network to construct the lncRNA-disease heterogeneous network for improving the predictive performance, we designed another method of IDHI-AVG by adopting the strategy of averaging three lncRNA similarity matrices of LncNet1, LncNet2 and LncNet3 to form the lncRNA integration network (i.e., LncINet), averaging of three disease similarity matrices of DisNet1, DisNet2, and DisNet3 to form the disease integration network (i.e., DisINet). IDHI-AVG combines these two integration similarity networks of LncINet and DisINet with known lncRNA-disease bipartite network to construct the lncRNA-disease heterogeneous network on which RWRH algorithm is implemented to predict the potential lncRNA-disease associations. The compared results of IDHI-AVG and IDHI-MIRW on the small-scale lncRNA-disease heterogeneous network (HNet_S_) and large-scale ncRNA-disease heterogeneous network (HNet_L_) in LOOCV test are shown in Table 3. We can see the AUC and AUPR values of IDHI-MIRW are higher than that of IHDI-AVG. These results demonstrate that the strategy of using RWR and PPMI to form lncRNA/disease topological similarity networks and further constructing the lncRNA-disease heterogeneous network is effective. It can improve the performance of predicting lncRNA-disease associations.

**Table 3:** Compared results of IDHI-MIRW and IDHI-AVG on the small-scale lncRNA-disease heterogeneous network and large-scale lncRNA-disease heterogeneous network in LOOCV test

	HNet_S_		HNet_L_	
	IDHI-AVG	IDHI-MIRW	IDHI-AVG	IDHI-MIRW
AUC	0.829	0.866	0.942	0.952
AUPR	0.238	0.318	0.317	0.350

### The effect of parameters

There are four main parameters in our method, which are the restart probability *α* in RWR, and the restart probability *β*, jumping probability *γ*, parameter *η* in RWRH. *η* is used to weight the importance of lncRNA topological similarity subnetwork and disease topological similarity subnetwork. To evaluate the effect of parameters, we implemented our IDHI-MIRW on HNet_L_ heterogeneous network in LOOCV test with different *α*, *β*, *γ*, and *η* values (varying from 0.1 to 0.9 with scale 0.1). Additional file 3 shows the AUC and AUPR values of IDHI-MIRW with different parameters. We can see that the performance of IDHI-MIRW is robust to the value of these four parameters. Additional file 4 presents the AUC and AUPR values of IDHI-MIRW on HNet_S_ heterogeneous network in LOOCV test. In this work, we selected *α* = 0.9, *γ* = 0.9, *η* = 0.2, and *β* = 0.6.

### Case studies and the potential lncRNA-disease associations analysis

We used breast cancer, stomach cancer, and colorectal cancer as the cases to predict their potential associated lncRNAs with our IDHI-MIRW. For a given disease, all known lncRNAs associated with this given disease were considered as the seed nodes, and other remaining lncRNAs (i.e., without known association with the given disease) were considered as the candidates associated with the given disease. By implementing our IDHI-MIRW algorithm on the large-scale lncRNA-disease heterogeneous network, and according to the lncRNA-disease associations ranking scores from large to small, we extract top 15 potential association lncRNAs for each cancer. These top potential association lncRNAs are listed in Additional files 5, 6, and 7.

For breast cancer which is one of most common cancers and the second leading cause of cancer death [49], 13 out of 15 potential association lncRNAs are supported by recent literatures. For example, Diego Chacon-Cortes et al. [50] investigated six SNPs (i.e. rs1888138, rs7336610, rs9589207, rs17735387, rs4248505, rs1428) in the lncRNA MIR17HG, and identified significant association between rs4248505 at the allele level and rs4248505/ rs7336610 at the haplotype level susceptibility to breast cancer, which means that lncRNA MIR17HG plays the main role in the pathophysiology of breast cancer. Fu et al. [51] found lncRNA SNHG1, SNORD28 and sno-miR-28 are all significantly upregulated in breast tumors. LncRNA can be used as the biomarkers and therapeutic targets in combatting breast cancer [52].

For stomach cancer (or gastric cancer) which is the third leading cause of cancer mortality in the world [53, 54], 11 out of 15 potential association lncRNAs can be supported by recent literatures. For example, Hu et al. [55] discovered that lncRNA CRNDE increases gastric cancer cell viability and promotes proliferation by targeting miR-145. Pan et al. [56] found that lncRNA DANCR is activated by SALL4 and promotes the proliferation and invasion of gastric cancer cells. Specially, lncRNA LINC01816 (also known as LOC100133985) associated with stomach cancer has been confirmed by Tian et al. [57]. LncRNA LINC01816 is down-regulated and might be protective factor in gastric cancer.

For colorectal cancer which is the third most commonly diagnosed cancer in males and the second in females [58], 12 out of 15 potential association lncRNAs can be supported by recent literatures. For example, Zhao et al. [59] found that lncRNA SNHG1 promotes cell proliferation by affecting P53 in colorectal cancer. Zhang et al. [60] found that lncRNA CYTOR (also known as LINC00152) down-regulated by miR-376c-3p restricts viability and promotes apoptosis of colorectal cancer cells.

**Fig. 3:**
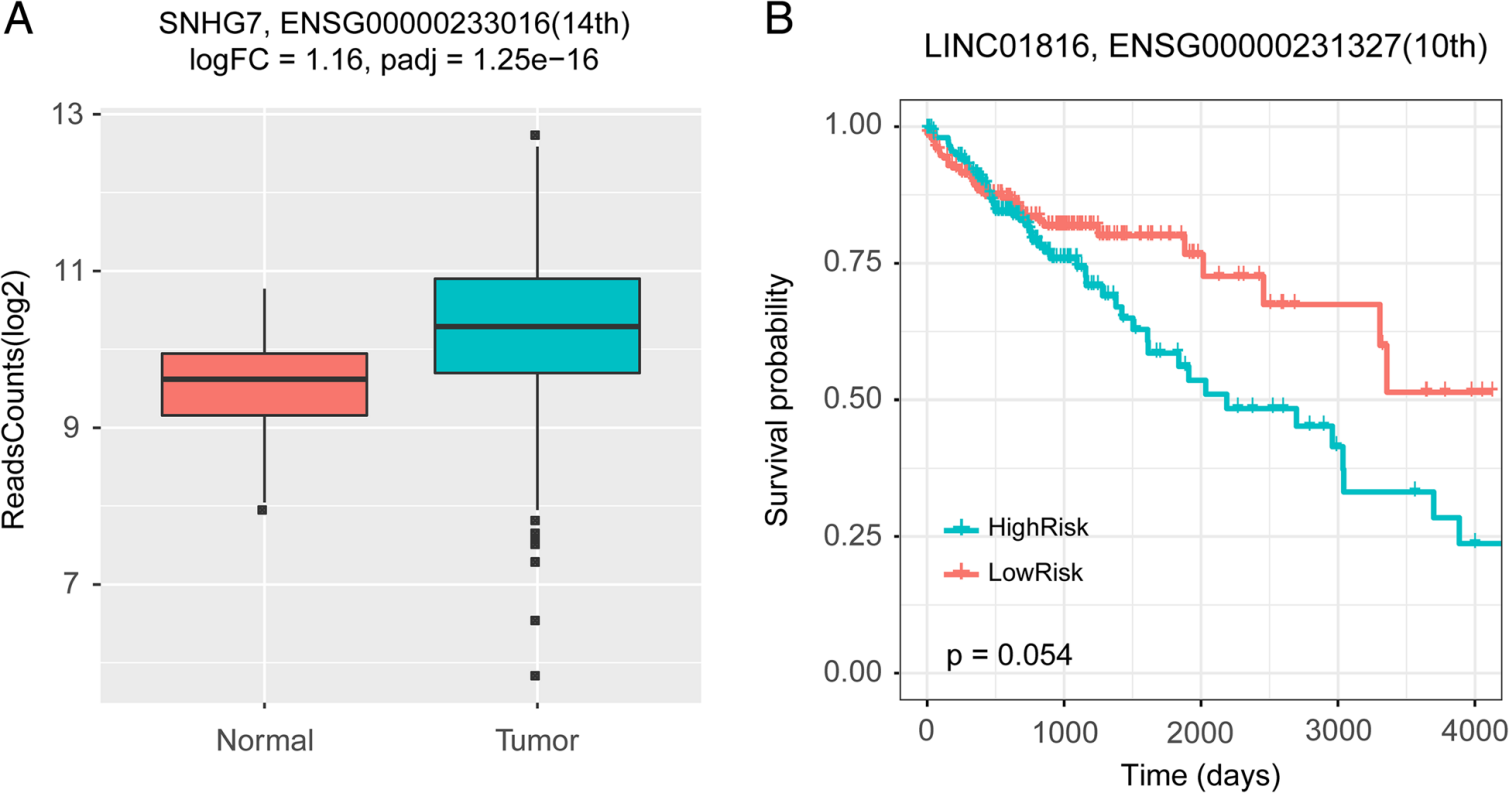
Results of RNASeq and clinical data analysis for colorectal cancer. **a** boxplot of lncRNA SNHG7 expression in normal and tumor samples. **b** survival curve for lncRNA LINC01816

To further discover the evidences for the predicted lncRNAs associated with cancers, we analyzed the RNAseq and clinical data from TCGA for breast cancer, stomach cancer and colorectal cancer. For colorectal cancer, the RNASeq data including 19,676 protein coding genes, 15,513 lncRNA genes in 41 normal samples and 474 tumor samples were downloaded from TCGA. Using DESeq2 [61] algorithm, we found 1230 significantly upregulated lncRNAs and 568 downregulated lncRNAs by setting log2FC > 1 (or < − 1), FDR < 0.001. Among three unvalidated lncRNA, lncRNA SNHG7 (14th) is significantly upregulated in tumor samples (Fig. 3a). Meanwhile, we downloaded the clinical data of 448 tumor samples, and Kaplan-Meier survival analysis shows that lncRNA LINC01816 (10th) can divided the 448 colorectal cancer patients into high and low-risk groups with different survival times (Fig. 3b). The results of RNAseq and clinical data analysis for breast cancer and stomach cancer are shown in.

Additional files 8 and 9. 5/6 unvalidated lncRNAs are significantly differentially expressed in corresponding cancers.

In summary, 36 (13 for breast cancer, 11 for stomach cancer, 12 for colorectal cancer) out of 45 potential association lncRNAs have been supported by recent literatures. By analyzing the nine unvalidated potential association lncRNAs, we found that six lncRNAs are differentially expressed in corresponding cancers, and lncRNA LINC01816 is associated with the survival of patients with colorectal cancer. Results of these three case studies show that IDHI-MIRW can effectively predict the new association lncRNAs for a disease.

## Discussion

LncRNAs play important roles in the development of human complex diseases. More and more attentions have been paid to discover the lncRNA functions related with human complex disease. Most previous computational methods only focus on the small-scale lncRNA-disease heterogeneous network (i.e., involving small numbers of lncRNAs and diseases) to predict the lncRNA-disease associations. To address this issue, IDHI-MIRW was developed to predict the potential lncRNA-disease associations based on a large-scale lncRNA-disease heterogeneous network (containing 7637 lncRNAs and 6453 diseases). Instead of calculating similarities of lncRNAs and diseases only involving in known lncRNA-disease associations, IDHI-MIRW used three lncRNA-related information (i.e., lncRNA expression profiles, lncRNA-miRNA interactions, and lncRNA-protein interactions) to form three lncRNA similarity networks, and three disease-related information (i.e., disease semantic similarity, disease-miRNA associations, and disease-gene associations) to form three disease similarity networks. Furthermore, instead of directly fusing those similarity networks, IDHI-MIRW applied the RWR algorithm on each lncRNA/disease similarity network to capture the topological similarity, and the PPMI to generate lncRNA/disease topological similarity network. The large-scale lncRNA-disease heterogeneous network was constructed by combing the lncRNA topological similarity network, disease topological similarity network, and the known lncRNA-disease bipartite graph. Then, the RWRH algorithm was used to prioritize candidate lncRNAs for each query disease. Our experiment results show that IDHI-MIRW achieves a better performance than other existing methods. We evaluated the effectiveness of introducing multiple information sources and capturing topological similarities, Tables 2 and 3 show that those strategies are effective for improving the performance of predicting lncRNA-disease associations. In addition, more novel lncRNA-disease associations predicted by IDHI-MIRW are supported by recent literatures, which means that IDHI-MIRW can effectively predict the novel association lncRNAs for a query disease. All the predicted lncRNA-disease associations are provided in Additional file 10.

Although IDHI-MIRW can effectively predict potential lncRNA-disease associations, there are still several issues need to be further addressed in the future. First, IDHI-MIRW used three lncRNA-related and three disease-related information to generate similarity matrices, we still expect to integrate more information (e.g., lncRNA GO annotations and disease MeSH annotation) to better predict lncRNA-disease association. Second, the averaging strategy was used to integrate the lncRNA/disease topological similarity matrices, we expect to design better integration approaches in future work to measure the different contributions of multiple lncRNA/disease similarities.

## Conclusions

In this study, we proposed a novel network-based method (namely IDHI-MIRW) for identifying potential lncRNA-disease associations. We built a large-scale lncRNA-disease heterogeneous network by integrating multiple lncRNA-related information (i.e. lncRNA expression profiles, lncRNA-miRNA interactions, and lncRNA-protein interactions), multiple disease-related information (i.e. disease semantic similarity, disease-miRNA associations, and disease-gene associations), and known lncRNA-disease association information using RWR and PPMI. Our experimental results show that IDHI-MIRW can achieve higher performance than other state-of-the-art methods, and we found lncRNA LINC01816 is associated with the survival of colorectal cancer patients. These results indicate that IDHI-MIRW will contribute to the identification of potential lncRNA-disease associations.

## Methods

### Datasets

We collected lncRNA expression profile, lncRNA-miRNA interaction, and lncRNA-protein interaction data for constructing the lncRNA similarity networks, and Diseases Ontology (DO) information, disease-miRNA association, and disease-protein association data for constructing the disease similarity networks. All lncRNAs are annotated by ensembl gene ID, and all diseases are annotated by Disease Ontology ID.

LncRNA expression profiles were downloaded from EMBL-EBI (E-MTAB-5214), which includes the expression profiles in 53 human tissue samples. LncRNA-miRNA interactions and lncRNA-protein interactions were collected from starBase v2.0 [43], NPInter v3.0 [44], and RAID v2.0 [45] databases. Diseases ontology terms were collected from the Disease ontology [46]. Diseases-miRNAs associations were collected from HMDD v2.0 [47]. Disease-gene associations were collected from DisGeNet [48]. Known lncRNA-disease associations were collected from lncRNAdisease [15], lnc2Cancer [16], and GeneRIF [62]. Details and statistics of these data are shown in Additional file 11.

### An overview of the IDHI-MIRW algorithm

**Fig. 4:**
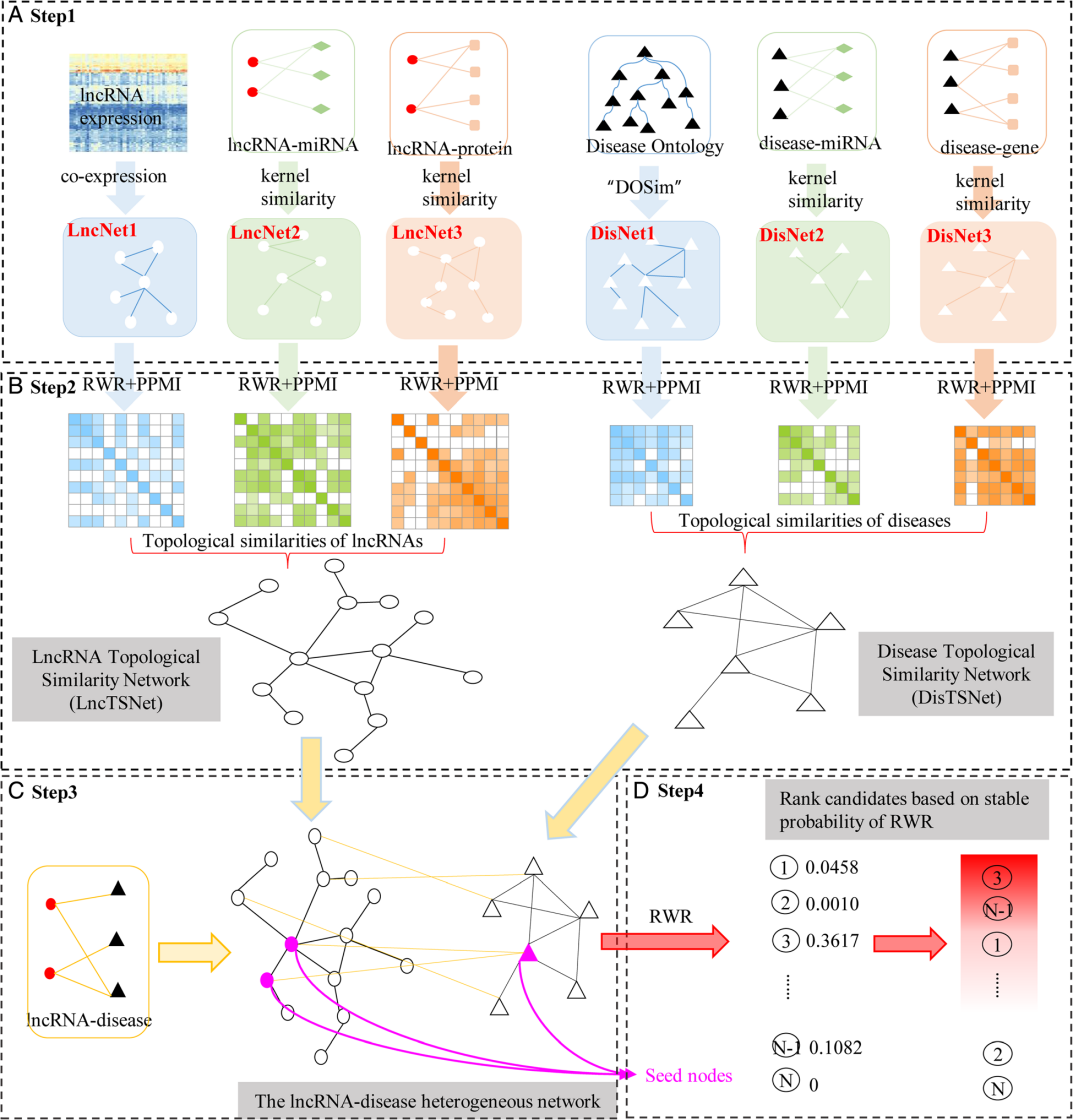
Flowchart of the IDHI-MIRW. **a** building three lncRNA similarity networks and three disease similarity networks by calculating the Pearson correlation coefficient and Gaussian interaction profile kernel similarity. **b** forming the lncRNA/disease topological similarity networks with RWR and positive pointwise mutual information. **c** constructing the large-scale lncRNA-disease heterogeneous network by integrating lncRNA/disease topological similarities and known lncRNA-disease associations. **d** predicting the potential lncRNA-disease associations by implementing RWRH

Our IDHI-MIRW algorithm consists of the following four steps. Step 1, build three lncRNA similarity networks (i.e., LncNet1, LncNet2, LncNet3) based on lncRNA expression profiles, lncRNA-miRNA interactions, and lncRNA-protein interactions, and also build three disease similarity networks (i.e., DisNet1, DisNet2, DisNet3) based on disease ontology, disease-miRNA associations, and disease-gene associations. Step 2, form the lncRNA topological similarity network (LncTSNet) and disease topological similarity network (DisTSNet) by fusing lncRNA and disease multiple topological similarities obtained through implementing RWR on lncRNA similarity network (LncNet1, LncNet2, LncNet3) and disease similarity network (DisNet1, DisNet2, DisNet3), respectively. Step 3, construct a large-scale lncRNA-disease heterogeneous network by integrating lncRNA topological similarity network (LncTSNet), disease topological similarity network (DisTSNet), and known lncRNA-disease associations. Step 4, implement RWRH on the lncRNA-disease heterogeneous network for predicting the potential lncRNA-disease associations. The flowchart of IDHI-MIRW is shown in Fig. 4.

### Building lncRNA/disease similarity networks

By calculating the Pearson correlation coefficient of any lncRNA pair with expression profiles and fixing the *P*-value threshold (< 0.01), we built the LncNet1 lncRNA similarity weighted network. Based on Gaussian interaction profile kernel similarity [18, 63] of lncRNA-miRNA and lncRNA-protein interactions, we computed the Gaussian interaction profile kernel similarity between any pair of lncRNA *l*_*i*_ and lncRNA *l*_*j*_, then built the LncNet2 and LncNet3 lncRNA similarity weighted networks, respectively. Gaussian interaction profile kernel similarity between lncRNA *l*_*i*_ and lncRNA *l*_*j*_ is calculated.

1 $$ KD\left({l}_i,{l}_j\right)=\mathrm{Exp}\left(-{\kappa}_l\left\Vert IP\left({l}_i\right)- IP\left({l}_j\right)\right\Vert \right) $$

2 $$ {\kappa}_l=1/\left(\frac{1}{N_l}\sum i=1{N}_l{\left\Vert IP\left({l}_i\right)\right\Vert}^2\right) $$

where, the interaction profile *IP*(*l*_*i*_) is the binary vector of lncRNA-miRNA (or lncRNA-protein) interactions encoding the presence or absence of interactions between lncRNA *l*_*i*_ and miRNA (or protein) in the lncRNA-miRNA (or lncRNA-protein) interaction dataset, *κ*_*l*_ controls the kernel bandwidth, and *N*_*l*_ is the total number of lncRNAs.

Based on the structure of a directed acyclic graph (DAG) in Disease Ontology, we used the function “doSim” form R package “DOSE” [64] to obtain the similarity between any disease pair, then built the DisNet1 disease similarity weighted network. Based on Gaussian interaction profile kernel similarity of disease-miRNA and disease-gene associations, we computed the Gaussian interaction profile kernel similarity between any pair of disease *d*_*i*_ and *d*_*j*_, then built the DisNet2 and DisNet3 disease similarity weighted networks, respectively.

3 $$ KD\left({d}_i,{d}_j\right)=\exp \left(-{\kappa}_d\left\Vert IP\left({d}_i\right)- IP\left({d}_j\right)\right\Vert \right) $$

4 $$ {\kappa}_d=1/\left(\frac{1}{N_d}\sum i=1{N}_d{\left\Vert IP\left({d}_i\right)\right\Vert}^2\right) $$

where, the interaction profile *IP*(*d*_*i*_) is the binary vector of disease-miRNA (or disease-gene) associations encoding the presence or absence of associations between *d*_*i*_ and miRNA (or gene) in the disease-miRNA (or disease-gene) association dataset. *κ*_*d*_ controls the kernel bandwidth, and *N*_*d*_ is the total number of diseases.

### Generating lncRNA/disease topological similarity networks

Instead of directly fusing six similarity networks (i.e., LncNet1, LncNet2, LncNet3, DisNet1, DisNet2, and DisNet3), we captured the network topological structural features by implementing the RWR algorithm on each similarity network. The RWR algorithm is a network diffusion algorithm, which has been extensively applied to analyze the complex biological network [65–69]. By considering both local and global topological connectivity patterns within network, the RWR algorithm can fully exploit the direct or indirect relation between nodes [65]. The RWR algorithm can be formulated as:

5 $$ {S}^{t+1}=\left(1-\alpha \right){S}^tW+\alpha {S}^0 $$

6 $$ W\left(i,j\right)=\frac{B\left(i,j\right)}{\sum_jB\left(i,j\right)} $$

where, *S*^*t*^ is the distribution matrix in which the (*i*, *j*)-th element denotes the distribution probability of node *j* being visited from node *i* after *t* iterations in the random walk process and *S*^0^ is the initial distribution matrix in which *S*^0^(*i*, *i*) = 1, *S*^0^(*i*, *j*) = 0, ∀*j* ≠ *i*. *α* is restart probability controlling the relative influence of local and global topological information. *B* is the weighted adjacency matrix of lncRNA (or disease).

When the L1 norm of Δ*S* = *S*^*t* + 1^ − *S*^*t*^is less than a small positive *ε* (we set *ε* = 10^−10^), we can obtain a stationary distribution matrix *S*, which was referred as the diffusion state of each node [70]. The element *S*(*i*, *j*) in diffusion state matrix *S* represents the probability of RWR starting node *i* and ending up at node *j* in equilibrium. When the diffusion states of two nodes are close, which suggests that they may have similar positions with respect to other nodes in the network and they probably share similar functions.

Motivated by Gligorijevic et.al. [69], we then calculated the topological similarity of each node pair by using PPMI, which is defined as:

7 $$ MI\left(i,j\right)=\max \left(0,{\log}_2\frac{S\left(i,j\right){\sum}_i{\sum}_jS\left(i,j\right)}{\sum_iS\left(i,j\right){\sum}_jS\left(i,j\right)}\right) $$

The matrix *MI* is a non-symmetric matrix, thus we use the average of *MI*(*i*, *j*) and *MI*(*j*, *i*) to represent the topological similarity of node *i* and node *j*. After obtaining three lncRNA topological similarity matrices ${X}_L^1$, ${X}_L^2$, ${X}_L^3$ of LncNet1, LncNet2, LncNet3, and three disease topological similarity matrices ${X}_D^1$, ${X}_D^2$, ${X}_D^3$ of DisNet1, DisNet2, DisNet3, we can form the integration lncRNA topological similarity matrix ${X}_L^{\prime }$ by averaging three lncRNA topological similarity matrices, and the disease topological similarity matrix ${X}_D^{\prime }$ by averaging three disease topological similarity matrices, that is, ${X}_L^{\prime }=\left({X}_L^1+{X}_L^2+{X}_L^3\right)/3$, ${X}_D^{\prime }=\left({X}_D^1+{X}_D^2+{X}_D^3\right)/3$. Thus, we generated the lncRNA topological similarity network LncTSNet, and disease topological similarity network DisTSNet.

### Constructing the lncRNA-disease heterogeneous network

By integrating the LncTSNet and DisTSNet networks with known lncRNA-disease bipartite network, we can construct the lncRNA-disease heterogeneous network whose adjacency matrix can be defined as:

8 $$ \mathrm{A}=\left[\begin{array}{cc}{A}_L& {A}_{LD}\\ {}{A}_{DL}& {A}_D\end{array}\right] $$

where, *A*_*L*_ and *A*_*D*_ represent the weighted adjacency matrices of LncTSNet and DisTSNet, respectively; *A*_*LD*_ is the adjacency matrix of the lncRNA-disease bipartite graph; *A*_*DL*_ represents the transpose of *A*_*LD*_. If there is association between lncRNA *i* and disease *j* in known lncRNA-disease associations, *A*_*LD*_(*i*, *j*) = 1, otherwise, *A*_*LD*_(*i*, *j*) = 0.

### Implementing RWRH algorithm for predicting lncRNA-disease associations

To predict the association between lncRNA and disease, we adopted the RWRH (random walk with restart on heterogeneous network) algorithm [42] to prioritize candidate lncRNAs associated with a given disease. The RWRH algorithm is well-known heterogeneous network-based algorithm to infer the gene-phenotype relationship. It can effectively capture the complementarity of two kinds of node within heterogeneous network, which is widely used to predict the association problem [42, 71, 72]. The RWRH algorithm on the lncRNA-disease heterogeneous network can be formulated as:

9 $$ {p}^{t+1}=\left(1-\upbeta \right){p}^t\mathrm{M}+\upbeta {p}^0 $$

where, *p*^*t*^ is a probability vector in which the *i*-th element holds the probability of finding the random walker at node *i* at step *t*; *β* ∈ (0, 1) is restart probability; *p*^0^ is the initial probability vector for lncRNA-disease heterogeneous network which is defined as $$ {p}^0=\left[\begin{array}{c}\eta \ast {u}_0\\ {}\left(1-\eta \right)\ast {v}_0\end{array}\right] $$. *u*_0_ and *v*_0_ represent the initial probability of LncTSNet and DisTSNet, respectively. The initial probability *u*_0_ of LncTSNet network is set such that all the seed nodes are assigned to the equal probabilities with the sum of probabilities equal to 1. Similarity, the initial probability *v*_0_ of DisTSNet network is given. The parameter *η* ∈ (0, 1) is used to weight the importance of each subnetwork.

$$ M=\left[\begin{array}{cc}{M}_L& {M}_{LD}\\ {}{M}_{DL}& {M}_D\end{array}\right] $$ is the transition matrix of the lncRNA-disease heterogenous network, where *M*_*L*_ and *M*_*D*_ are the intra-subnetwork transition matrices, *M*_*LD*_ and *M*_*DL*_ are the inter-subnetwork transition matrices. Let *γ* be the jumping probability, that is, the probability of random walker jumping from lncRNA network to disease network or vice versa. Thus, the transition probability *M*_*L*_(*i*, *j*) from lncRNA *l*_*i*_ to lncRNA *l*_*j*_ and the transition probability *M*_*D*_ (*i*, *j*) from disease *d*_*i*_ to disease *d*_*j*_ are defined as

10 $$ {M}_L\left(i,j\right)=\left\{\begin{array}{c}\raisebox{1ex}{${A}_L\left(i,j\right)$}\!\left/ \!\raisebox{-1ex}{${\sum}_j\ {A}_L\left(i,j\right)$}\right.\  if{\sum}_j\ {A}_{LD}\left(j,i\right)=0\\ {}\raisebox{1ex}{$\left(1-\gamma \right){A}_L\left(i,j\right)$}\!\left/ \!\raisebox{-1ex}{${\sum}_j\ {A}_L\left(i,j\right)$}\right.\  otherwise\end{array}\right. $$

11 $$ {M}_D\left(i,j\right)=\left\{\begin{array}{c}\raisebox{1ex}{${A}_D\left(i,j\right)$}\!\left/ \!\raisebox{-1ex}{${\sum}_j\ {A}_D\left(i,j\right)$}\right.\  if\ {\sum}_j\ {A}_{LD}\left(i,j\right)=0\\ {}\raisebox{1ex}{$\left(1-\gamma \right){A}_D\left(i,j\right)$}\!\left/ \!\raisebox{-1ex}{${\sum}_j\ {A}_D\left(i,j\right)$}\right.\  otherwise\end{array}\right. $$

The transition probability from lncRNA *l*_*i*_ to disease *d*_*j*_ and the transition probability from disease *d*_*i*_ to lncRNA *l*_*j*_ are described as:

12 $$ {M}_{LD}\left(i,j\right)=\left\{\begin{array}{c}\raisebox{1ex}{${\gamma A}_{LD}\left(i,j\right)$}\!\left/ \!\raisebox{-1ex}{${\sum}_j\ {A}_{LD}\left(i,j\right)$}\right. if\ {\sum}_j\ {A}_{LD}\left(i,j\right)\ne 0\\ {}0\  otherwise\end{array}\right. $$

13 $$ {M}_{DL}\left(i,j\right)=\left\{\begin{array}{c}\raisebox{1ex}{${\gamma A}_{DL}\left(i,j\right)$}\!\left/ \!\raisebox{-1ex}{${\sum}_j\ {A}_{DL}\left(i,j\right)$}\right.\  if\ {\sum}_j\ {A}_{DL}\left(i,j\right)\ne 0\\ {}0\  otherwise\end{array}\right. $$

After some steps, the steady state probability vector *p*^∗^ = *p*^∞^ can be obtained by performing the iteration until the difference between *p*^*t*^ and *p*^*t* + 1^ (measured by the L_1_ norm) fall below 10^−10^. *p*^∗^ gives the ranking score of every lncRNA for a query disease. The lncRNAs with maximum in *p*^∗^ are considered as the most probable associated lncRNAs of the query disease.

## Additional files

Additional file 1: LncRNA data processing procedure. (TIF 1447 kb)

Additional file 2: Disease data processing procedure. (TIF 1340 kb)

Additional file 3: AUPR values of IDHI-MIRW on the large-scale lncRNA-disease heterogeneous with different parameters in LOOCV test. (A) AUC values with different *α*. (B) AUC values with different *γ*. (C) AUC values with different *η*. (D) AUC values with different *β*. (E) AUPR values with different *α*. (F) AUPR values with different *γ*. (G) AUPR values with different *η*. (H) AUPR values with different *β*. (TIF 3520 kb)

Additional file 4: AUC and AUPR values of IDHI-MIRW on the small-scale lncRNA-disease heterogeneous with different parameters in LOOCV test. (A) AUC values with different *α*. (B) AUC values with different *γ*. (C) AUC values with different *η*. (D) AUC values with different *β*. (E) AUPR values with different *α*. (F) AUPR values with different *γ*. (G) AUPR values with different *η*. (H) AUPR values with different *β*. (TIF 3705 kb)

Additional file 5: The top 15 predicted associated lncRNAs for breast cancer. (XLSX 9 kb)

Additional file 6: The top 15 predicted associated lncRNAs for stomach cancer. (XLSX 9 kb)

Additional file 7: The top 15 predicted associated lncRNAs for colorectal cancer. (XLSX 9 kb)

Additional file 8: The results of RNASeq data analysis for breast cancer. (A) heatmap of top 200 most significantly dysregulated lncRNA expression values. (B) heatmap of lncRNA AL157395.1 expression values. (C) boxplot of lncRNA AL157395.1 expression in normal and tumor samples. (D) heatmap of lncRNA AP001528.1 expression values. (E) boxplot of lncRNA AP001528.1 expression in normal and tumor samples. (TIF 9850 kb)

Additional file 9 The results of RNASeq data analysis for stomach cancer. (A) heatmap of top 200 most significantly dysregulated lncRNA expression values. (B) heatmap of lncRNA KCNQ1OT1 expression values. (C) boxplot of lncRNA KCNQ1OT1 expression in normal and tumor samples. (D) heatmap of lncRNA DLEU2 expression values. (E) boxplot of lncRNA DLEU2 expression in normal and tumor samples. (F) heatmap of lncRNA LINC00299 expression values. (G) boxplot of lncRNA LINC00299 expression in normal and tumor samples. (TIF 9211 kb)

Additional file 10: The predicted lncRNA-disease associations. (TXT 180 kb)

Additional file 11: Details and statistics of collected data. (DOCX 34 kb)

## Abbreviations

AUC: The area under the receiver operating characteristic curve
AUPR: The area under the precision-recall curve
DAG: Directed acyclic graph
DO: Disease ontology
FPR: False-positive rate
lncRNAs: Long noncoding RNAs
LOOCV: Leave-one-out cross validation; ROC: receiver operating characteristic
PPMI: Positive pointwise mutual information
PR: Precision-recall
RWR: Random walk with restart
RWRH: Random walk with restart on heterogeneous network
TPR: True-positive rate
