# Supplementary figures and images for: Prediction of lncRNA-disease associations by integrating diverse heterogeneous information sources with RWR algorithm and positive pointwise mutual information
Source: BMC Bioinformatics. 2019 Feb 19;20:87. doi: 10.1186/s12859-019-2675-y (PMC6381749; doi:10.1186/s12859-019-2675-y)

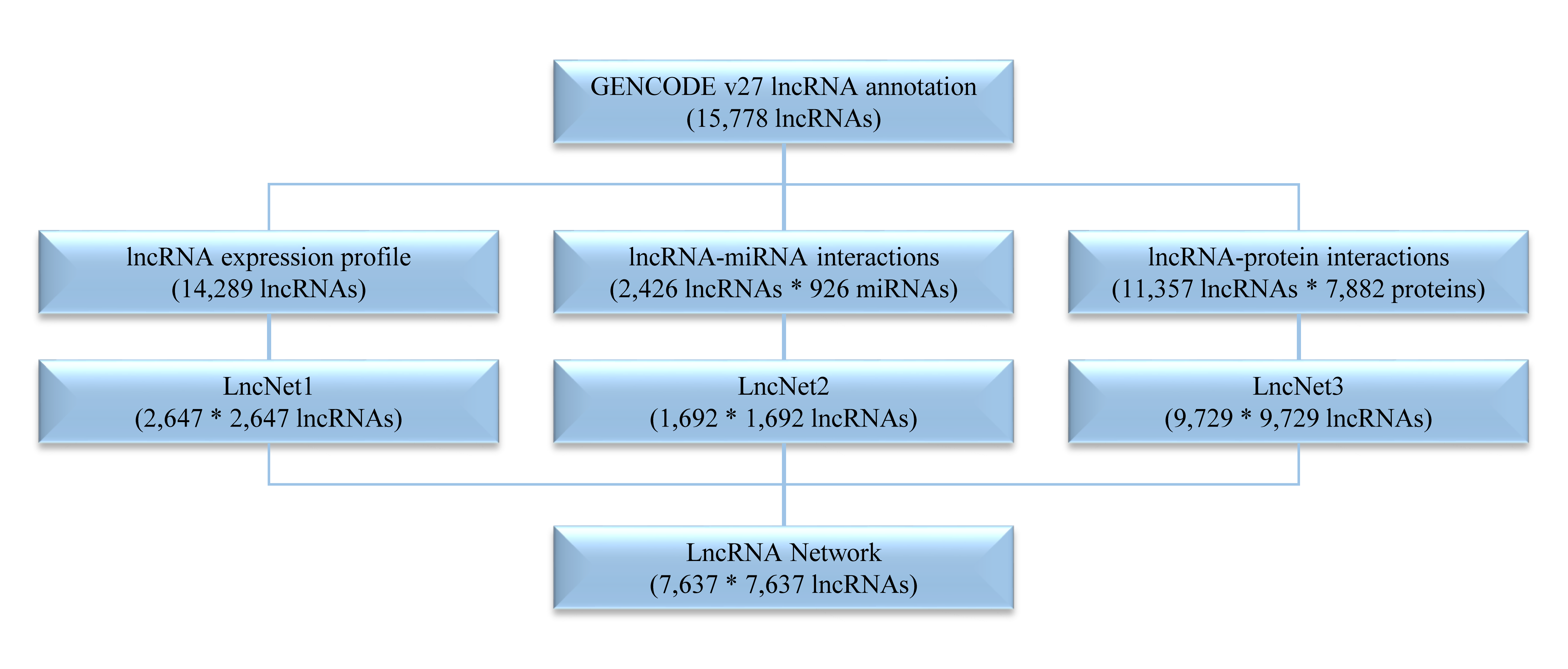

Supplement: Supplementary file 1 — LncRNA data processing procedure. (TIF 1447 kb) [file 12859_2019_2675_MOESM1_ESM.tif]

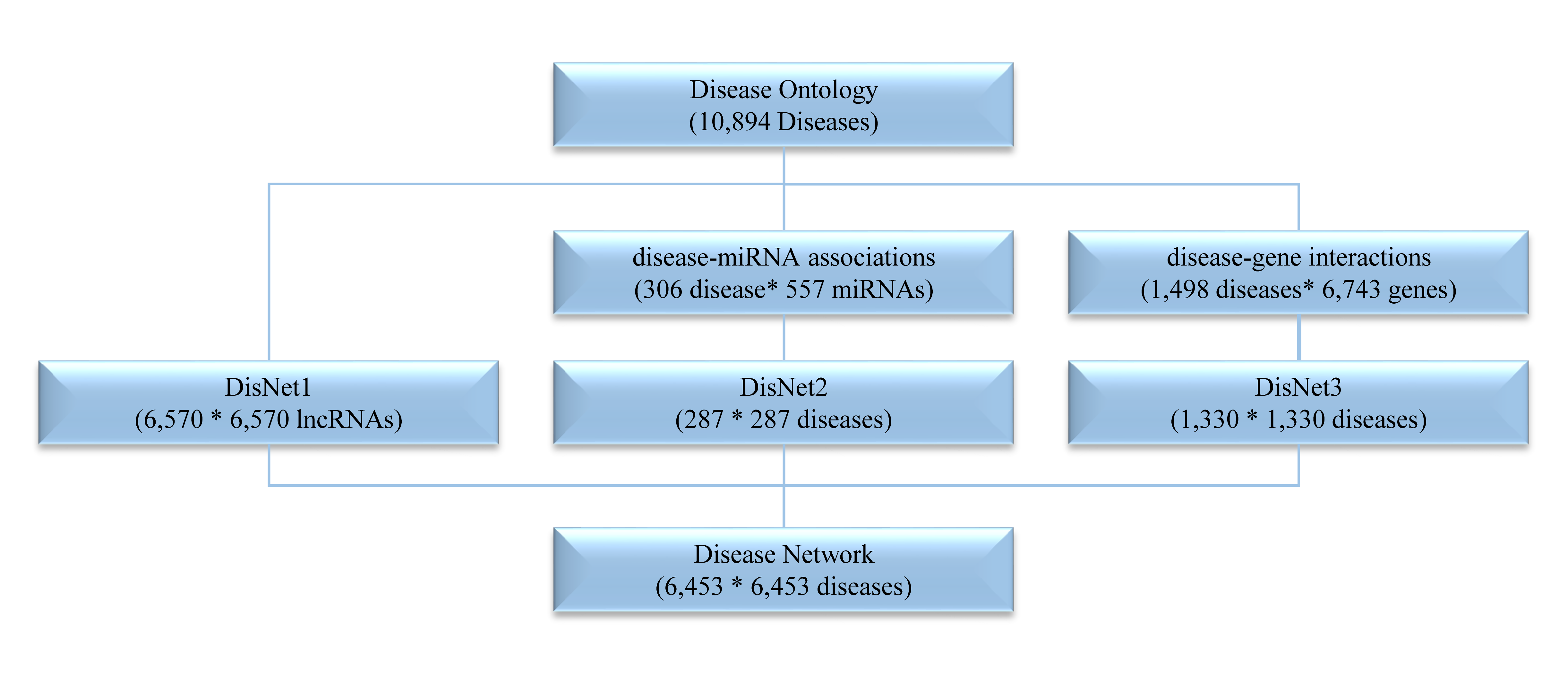

Supplement: Supplementary file 2 — Disease data processing procedure. (TIF 1340 kb) [file 12859_2019_2675_MOESM2_ESM.tif]

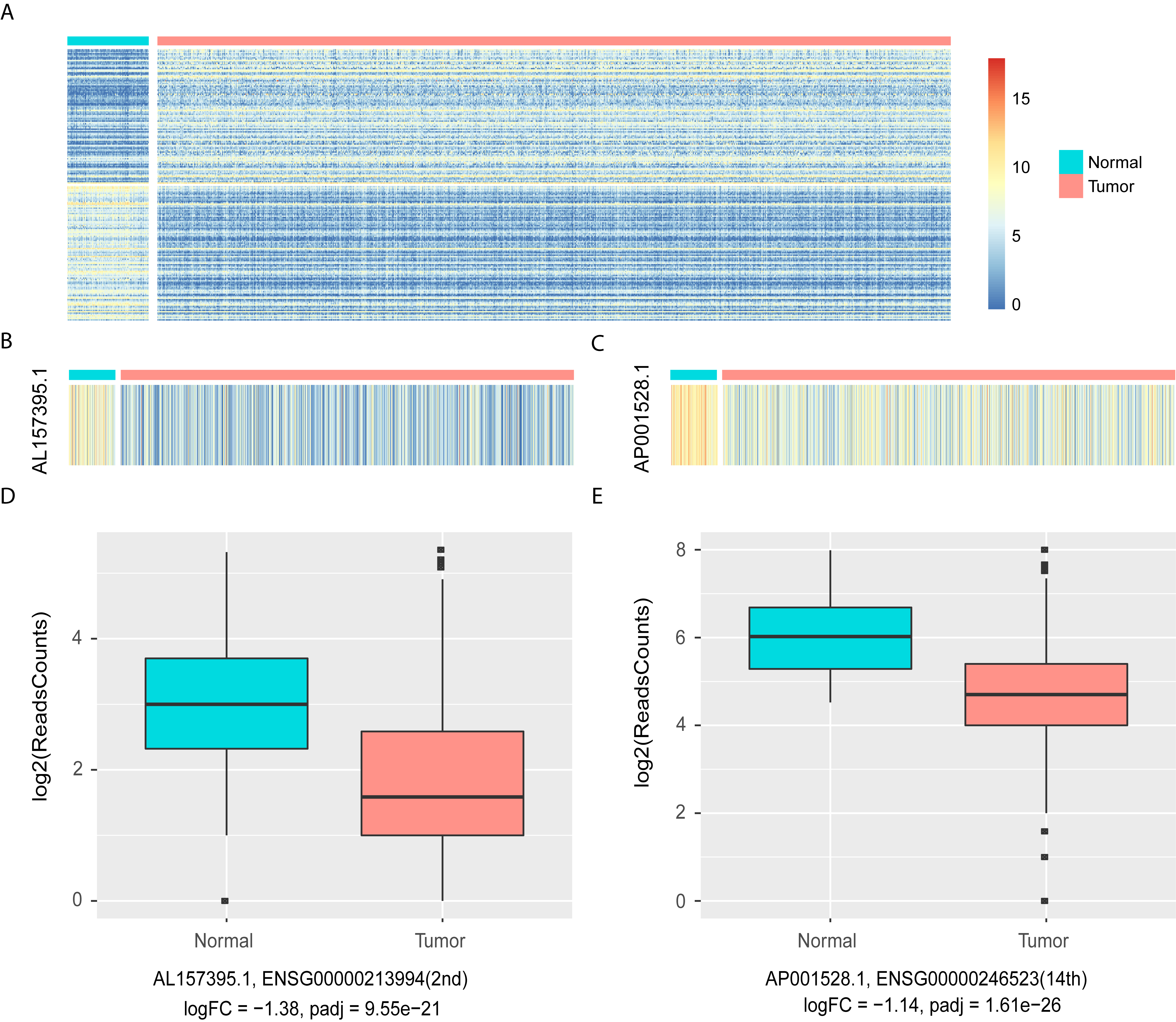

Supplement: Supplementary file 8 — The results of RNASeq data analysis for breast cancer. (A) heatmap of top 200 most significantly dysregulated lncRNA expression values. (B) heatmap of lncRNA AL157395.1 expression values. (C) boxplot of lncRNA AL157395.1 expression in normal and tumor samples. (D) heatmap of lncRNA AP001528.1 expression values. (E) boxplot of lncRNA AP001528.1 expression in normal and tumor samples. (TIF 9850 kb) [file 12859_2019_2675_MOESM8_ESM.tif]

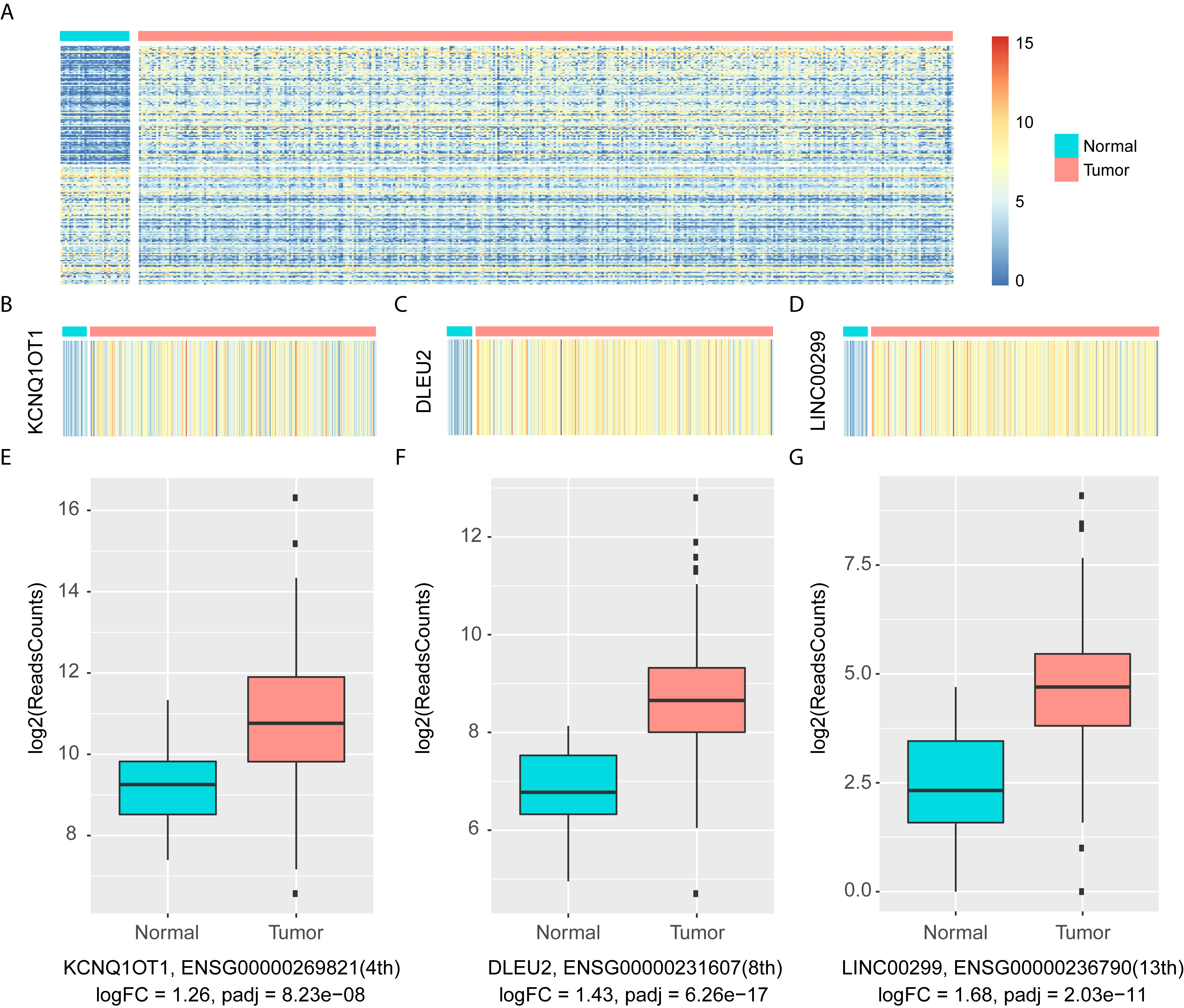

Supplement: Supplementary file 9 — The results of RNASeq data analysis for stomach cancer. (A) heatmap of top 200 most significantly dysregulated lncRNA expression values. (B) heatmap of lncRNA KCNQ1OT1 expression values. (C) boxplot of lncRNA KCNQ1OT1 expression in normal and tumor samples. (D) heatmap of lncRNA DLEU2 expression values. (E) boxplot of lncRNA DLEU2 expression in normal and tumor samples. (F) heatmap of lncRNA LINC00299 expression values. (G) boxplot of lncRNA LINC00299 expression in normal and tumor samples. (TIF 9211 kb) [file 12859_2019_2675_MOESM9_ESM.tif]
