# Supplementary material for: Prediction of lncRNA-disease associations by integrating diverse heterogeneous information sources with RWR algorithm and positive pointwise mutual information
Source: BMC Bioinformatics. 2019 Feb 19;20:87. doi: 10.1186/s12859-019-2675-y (PMC6381749; doi:10.1186/s12859-019-2675-y)
Supplement: Supplementary file 11 — Details and statistics of collected data. (DOCX 34 kb) [file 12859_2019_2675_MOESM11_ESM.docx]

**Data collections**

LncRNA expression profiles were downloaded from EMBL-EBI [1] (E-MTAB-5214, <https://www.ebi.ac.uk/arrayexpress/experiments/E-MTAB-5214/>). LncRNA-protein/lncRNA-miRNA interactions were collected from starBase v2.0 [2], NPInter v3.0 [3] and RAID v2.0 [4]. We firstly downloaded all the interactions from these three databases and deleted repeated entries, then the interactions containing lncRNA without ensembl gene ID are deleted. We finally selected the human lncRNA-protein/lncRNA-miRNA interactions with evidences from scientific literatures and high-throughput experimental technologies.

Diseases ontology terms were extracted from the disease ontology [5]. Human disease-miRNA associations and disease-gene associations were downloaded from HMDD v2.0 [6] and DisGeNet [7], respectively. Then, the entries containing disease without diseases ontology ID are deleted.

Known lncRNA-disease associations were collected from lncRNAdisease [8], lnc2Cancer [9], and GeneRIF [10]. We merged all the lncRNA-disease associations together without repeated entries, then all lncRNAs are annotated by ensembl gene ID, and all diseases are annotated by disease ontology ID. As a result, 2,169 lncRNA-disease associations were obtained, which were used as the golden standard dataset in our study.

Table S1. Statistics of collected data

| Name | Samples | #Associations | Sources |
| --- | --- | --- | --- |
| lncRNA expression profiles | 14,289 * 53 | - | E-MTAB-5214 [1] |
| lncRNA-miRNA | 2,426 * 926 | 28,301 | starBase v2.0 [2]  NPInter v3.0 [3]  RAID v2.0 [4] |
| lncRNA-protein | 11,357 * 7,882 | 75,918 | starBase v2.0 [2]  NPInter v3.0 [3]  RAID v2.0 [4] |
| Disease Ontology | 6,570 | - | Disease Ontology [5] |
| Disease-miRNA | 306 * 557 | 6,234 | HMDD v2.0 [6] |
| Disease-gene | 1,498 * 6,743 | 38,796 | DisGeNet [7] |
| lncRNA-disease | 362 * 370 | 2,169 | lncRNAdisease [8]  Lnc2Cancer [9]  GeneRIF [10] |

**Supplemental references:**

1. **EMBL-EBI** [<https://www.ebi.ac.uk/>]

2. Li JH, Liu S, Zhou H, Qu LH, Yang JH: **starBase v2.0: decoding miRNA-ceRNA, miRNA-ncRNA and protein-RNA interaction networks from large-scale CLIP-Seq data**. *Nucleic Acids Res* 2014, **42**(Database issue):D92-97.

3. Hao Y, Wu W, Li H, Yuan J, Luo J, Zhao Y, Chen R: **NPInter v3.0: an upgraded database of noncoding RNA-associated interactions**. *Database (Oxford)* 2016, **2016**.

4. Yi Y, Zhao Y, Li C, Zhang L, Huang H, Li Y, Liu L, Hou P, Cui T, Tan P *et al*: **RAID v2.0: an updated resource of RNA-associated interactions across organisms**. *Nucleic Acids Res* 2017, **45**(D1):D115-D118.

5. Schriml LM, Arze C, Nadendla S, Chang YW, Mazaitis M, Felix V, Feng G, Kibbe WA: **Disease Ontology: a backbone for disease semantic integration**. *Nucleic Acids Res* 2012, **40**(Database issue):D940-946.

6. Li Y, Qiu C, Tu J, Geng B, Yang J, Jiang T, Cui Q: **HMDD v2.0: a database for experimentally supported human microRNA and disease associations**. *Nucleic Acids Res* 2014, **42**(Database issue):D1070-1074.

7. Pinero J, Bravo A, Queralt-Rosinach N, Gutierrez-Sacristan A, Deu-Pons J, Centeno E, Garcia-Garcia J, Sanz F, Furlong LI: **DisGeNET: a comprehensive platform integrating information on human disease-associated genes and variants**. *Nucleic Acids Res* 2017, **45**(D1):D833-D839.

8. Chen G, Wang Z, Wang D, Qiu C, Liu M, Chen X, Zhang Q, Yan G, Cui Q: **LncRNADisease: a database for long-non-coding RNA-associated diseases**. *Nucleic Acids Res* 2013, **41**(Database issue):D983-986.

9. Ning S, Zhang J, Wang P, Zhi H, Wang J, Liu Y, Gao Y, Guo M, Yue M, Wang L *et al*: **Lnc2Cancer: a manually curated database of experimentally supported lncRNAs associated with various human cancers**. *Nucleic Acids Res* 2016, **44**(D1):D980-985.

10. Lu Z, Cohen KB, Hunter L: **GeneRIF quality assurance as summary revision**. *Pac Symp Biocomput* 2007:269-280.
